# Pathophysiology and therapeutic potential of cardiac fibrosis

**DOI:** 10.1186/s41232-017-0046-5

**Published:** 2017-07-17

**Authors:** Hironori Hara, Norifumi Takeda, Issei Komuro

**Affiliations:** Department of Cardiovascular Medicine, The University of Tokyo Hospital, 7-3-1 Hongo, Bunkyo-ku, Tokyo 113-8655 Japan

**Keywords:** Cardiomyocytes, Cardiac fibroblasts, Cardiac regeneration, Direct reprogramming, Angiogenesis

## Abstract

Inflammatory and fibrotic responses to myocardial damage are essential for cardiac repair; however, these responses often result in extensive fibrotic remodeling with impaired systolic function. Recent reports have suggested that such acute phase responses provide a favorable environment for endogenous cardiac regeneration, which is mainly driven by the division of pre-existing cardiomyocytes (CMs). Existing CMs in mammals can re-acquire proliferative activity after substantial cardiac damage, and elements other than CMs in the physiological and/or pathological environment, such as hypoxia, angiogenesis, and the polarity of infiltrating macrophages, have been reported to regulate replication. Cardiac fibroblasts comprise the largest cell population in terms of cell number in the myocardium, and they play crucial roles in the proliferation and protection of CMs. The in vivo direct reprogramming of functional CMs has been investigated in cardiac regeneration. Currently, growth factors, transcription factors, microRNAs, and small molecules promoting the regeneration and protection of these CMs have also been actively researched. Here, we summarize and discuss current studies on the relationship between cardiac inflammation and fibrosis, and cardiac regeneration and protection, which would be useful for the development of therapeutic strategies to treat and prevent advanced heart failure.

## Background

The number of deaths from cardiovascular diseases is increasing globally, and cardiac dysfunction is closely associated with increased myocardial fibrosis and loss of cardiomyocytes (CMs). Although cardiac fibrosis plays an essential role in the response to pressure overload and/or cardiac injury such as myocardial infarction (MI), its excessive and prolonged reaction can lead to cardiac diastolic and systolic dysfunction. Therefore, the regulation of inflammation and fibrosis at the appropriate timing and duration is crucial for the preservation or recovery of cardiovascular homeostasis. Currently, the inhibition of the renin–angiotensin system (RAS) using angiotensin-converting enzyme (ACE) inhibitors and angiotensin receptor blockers (ARBs) is the most validated clinical strategy for treating patients with advanced heart failure [1].

**Fig. 1:**
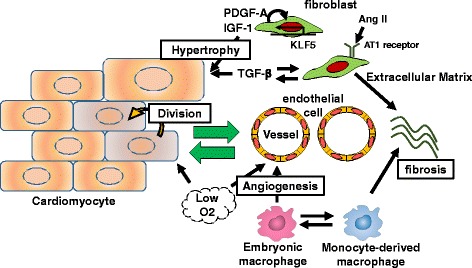
Interactions among cardiac cells. Most types of cardiac cells, including CMs, cardiac fibroblasts, macrophages, and endothelial cells, regulate cardiac fibrosis and regeneration in a coordinated manner. Some paracrine factors from fibroblasts, including TGF-β and IGF-1, are known to promote the hypertrophic responses of CMs. The regulation of hypoxic environment and macrophage polarization is a key factor for enhancing crucial angiogenic responses involved in cardiac repair and regeneration

Cardiac fibroblasts comprise the largest cell population in the myocardium [2], in terms of cell number, and they play a major role in fibrosis by producing the extracellular matrix (ECM) [3]. Cardiac fibroblasts interact with not only CMs but also with non-CMs, including vascular endothelial cells, smooth muscle cells, and immune cells, via direct and indirect cellular communications in an autocrine or paracrine manner [4] (Fig. 1). Recently, cardiac inflammation and fibrosis have been reported to be associated with the cardiac regenerative ability, which is mainly driven by the division of pre-existing CMs [5]; therefore, the modulation of the function of non-MCs for cardiac protection and regeneration has been actively investigated.

Here, we summarize and discuss current studies on the relationship between cardiac inflammation and fibrosis, and cardiac regeneration and protection, which would be useful for the development of therapeutic strategies for treating patients with advanced heart failure.

## Main text

### Types of cardiac fibrosis

Cardiac fibrosis is classified into two types: reactive fibrosis and reparative (replacement) fibrosis. Reactive fibrosis, which is characterized by the excessive deposition of ECM in the interstitial or perivascular spaces, is triggered by hemodynamic stress, such as pressure overload, and it is not directly associated with CM death [6, 7]. Reactive fibrosis is considered an adaptive response aimed at normalizing the increased wall stress and preserving the cardiac output. However, excessive fibrosis in interstitial spaces may cause mechanical stiffness, resulting in cardiac diastolic dysfunction, and impairment in electric conduction by forming a barrier between CMs, leading to cardiac systolic dysfunction. In addition, excessive fibrosis in perivascular areas decreases the flow of oxygen and nutrients, leading to an energy-starved condition in the myocardium [3]. Therefore, reactive cardiac fibrosis is closely associated with physiological and pathological cardiac conditions. Reparative fibrosis, which occurs in response to the loss of viable myocardium and forms a scar, maintains the structural integrity of the ventricles. A balance between reactive and reparative fibrosis is important for the prevention of excessive and inappropriate cardiac dysfunction, particularly after CM death due to cardiac injury, such as MI [8].

### Cardiac fibroblasts

Cardiac fibroblasts are flat, spindle-shaped cells located in the myocardium, with multiple processes originating from the cell body, and lack a basement membrane [3]. They play a major role in cardiac fibrosis by producing the ECM [3], and recent studies have demonstrated that mouse cardiac resident fibroblasts derived from the cells of the embryonic proepicardial organ (PEO) [9, 10] are major cell type producing the fibrotic ECM in a pressure overload model [11, 12]. However, other cell types have also been reported as origins of cardiac fibroblasts such as embryonic endothelium, which undergo endothelial-to-mesenchymal transition (EndMT) [13], circulating bone marrow cells [14], pericytes, and endothelial cells [15]. Because these cardiac fibroblasts lack a specific marker [11, 16, 17], investigating their regulation remains a challenging task.

### Paracrine factors associated with cardiac fibrosis

Transforming growth factor-beta (TGF-β) and angiotensin II (Ang II) are major factors that regulate cardiac fibrosis (Fig. 1). The expression of the Ang II type 1 (AT1) receptor is greater in fibroblasts than in CMs [18]. The activation of the AT1 receptor in fibroblasts by Ang II leads to the secretion of TGF-β, which stimulates fibroblast proliferation and ECM protein synthesis in an autocrine manner [19, 20] and induces CM hypertrophy in a paracrine manner [18]. The infusion of a subpressor dose of Ang II into mice induces both cardiac hypertrophy and fibrosis [21]. Clinical studies have demonstrated that the blockade of RAS signaling by an ACE inhibitor or ARB effectively reduces cardiac fibrosis and remodeling and that this is independent of the blood pressure-lowering effect [22]. However, the concomitant use of aliskiren, the direct renin inhibitor, with an ACE inhibitor or ARB in post-MI patients with reduced left ventricular (LV) ejection fraction does not further attenuate LV remodeling but is instead associated with more adverse effects [23]. The effect of blocking RAS signaling for cardiac fibrosis may eventually reach a plateau, with an excessive RAS blockade increasing adverse effects. Therefore, the appropriate regulation of RAS signaling is important for the prevention of cardiac fibrosis without any adverse effects.

TGF-β plays an essential role in cardiac fibrosis. Treatment with a subpressor dose of Ang II does not induce cardiac hypertrophy or fibrosis in *Tgfb1*-deficient mice [24]. Therefore, Ang II-induced cardiac fibrosis is believed to be mediated, at least in part, by TGF-β. Although cardiac hypertrophy and fibrosis induced by TGF-β signaling are adaptive responses to acute stress [3], the inhibition of TGF-β signaling may be useful for treating cardiac fibrosis. Therapies targeting TGF-β signaling have already been investigated in various mammalian models. An intraperitoneal injection of a TGF-β neutralizing antibody into rats subjected to pressure overload not only inhibits fibroblast activation and cardiac fibrosis but also prevents diastolic dysfunction [25]. In contrast, in a mouse aortic banding-induced pressure overload model, an orally active, small molecule inhibitor of the TGF-β type I receptor (TGFBR1, also known as activin receptor-like kinase 5), SM16, attenuates the development of cardiac fibrosis but causes death because of rupture at the site of aortic banding [26]. Further studies using other models of hypertension-induced cardiac fibrosis, which are independent of aortic banding, should be conducted. An MI model has been used to evaluate the effects on cardiac fibrosis and function. The treatment of rats with GW788388, another orally active TGFBR1 inhibitor, 1 week after MI, significantly reduces TGF-β signaling and attenuates LV remodeling and systolic dysfunction [27]. However, an intraperitoneal injection of a TGF-β neutralizing antibody started either 1 week before or 5 days after MI increases mortality and exacerbates LV dilatation and contractile dysfunction in mice [28]. These results indicate that the consequences of inhibiting TGF-β are variable, depending on the disease model and the timing of inhibition, presumably because TGF-β signaling in the heart during stress plays different roles during the early and late phases of cardiovascular disease.

### Cardiac hypertrophy induced by cardiac fibroblasts

**Fig. 2:**
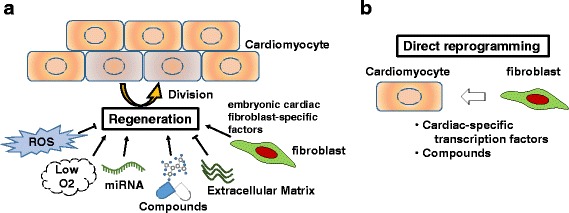
Current strategies for cardiomyocyte regeneration. **a** Endogenous cardiac regeneration is primarily driven by the division of pre-existing CMs; currently, paracrine factors, the microenvironment, and small molecules that regulate this process are under investigation. **b** The direct reprogramming of cardiac fibroblasts into CMs is induced by a combination of cardiac-specific transcription factors and compounds. Investigations to improve the efficiency and maturity of generated CMs are currently in progress

Some paracrine factors from cardiac fibroblasts induce CM proliferation and/or hypertrophy. Embryonic, but not adult, cardiac fibroblasts secrete high levels of fibronectin, collagen III, and heparin-binding EGF-like growth factor in mice. These embryonic cardiac fibroblast-specific factors collaboratively interact and promote embryonic CM proliferation (Fig. 2) [29]. On the other hand, in adult mice, various paracrine factors secreted by cardiac fibroblasts, including TGF-β, induce CM hypertrophy but not proliferation; the Krüppel-like factor 5 (KLF5) transcription factor expressed in adult cardiac fibroblasts promotes CM hypertrophy and cardiac protection (Fig. 1). KLF5 transactivates the expression of platelet-derived growth factor A (PDGF-A), which leads to the migration and proliferation of fibroblasts in an autocrine manner. Further, KLF5 transactivates insulin-like growth factor-1 (IGF-1) to promote CM hypertrophy in a paracrine manner. The cardiac fibroblast-specific deletion of *Klf5* ameliorates cardiac hypertrophy and fibrosis elicited by a moderate-intensity pressure overload [30]. On the other hand, a high-intensity pressure overload causes severe heart failure and early death in these mice. Furthermore, in wild-type mice, the administration of a peptide inhibitor of IGF-1 severely exacerbates heart failure induced by a high-intensity pressure overload. These results demonstrate that cardiac fibroblasts play pivotal roles in cardiac adaptive responses to pressure overload, which are, at least in part, regulated by IGF-1.

### Cellular sources of cardiac regeneration

The regenerative capacity greatly differs in adult mammalian organs, and organ-specific stem cells have been shown to contribute to regeneration in certain organs, such as the intestines, lungs, taste buds, and hair follicles [31–34]. In the mammalian heart, CMs rapidly proliferate during embryonic development; however, CMs exit the cell cycle, with the number of binucleated CMs increasing soon after birth [35]. One-day-old mice retain an adequate CM proliferative capacity and can completely regenerate CMs after cardiac injury, such as apical resection and MI. In response to cardiac injury, inflammation causes the proliferation of myofibroblasts and increases fibrosis in the regenerative area, but the myocardium is finally regenerated without fibrosis. Therefore, cardiac fibroblast-rich scar tissue may be an important component of cardiac repair in neonatal mice [17]. However, this efficient regenerative potential is lost within the first week of postnatal life [36, 37]; adult mice do not regenerate CMs adequately to compensate for the impaired cardiac function, inducing reparative fibrosis after injury instead. On the other hand, in certain lower vertebrates, such as teleost fish and urodele amphibians, adult CMs have sufficient regenerative capacity, and the myocardium can completely regenerate after injury without forming scar tissue [38, 39]. It is not known what causes these differences in the regenerative capacity of CMs between adult mammals and lower vertebrates. The fact that CMs in lower vertebrates are mononucleated and smaller in size with fewer myofibrils than those in adult mammals may be responsible for the differences observed in the CM regenerative capacity between these groups [40].

Recently, it has been shown that new CMs in adult humans are generated throughout life at a low rate (0.5–1% per year) [41]. Additional lines of evidence support the fact that the regeneration of adult mammalian CMs occurs at a low rate, decreasing with age but increasing with injury [42]. Various cell sources of endogenously regenerated CMs, such as pre-existing CMs, cardiac progenitor cells (CPCs), and cardiac fibroblasts, have been proposed, and lineage-tracing analyses (fate map) and/or cell transplantation studies have been used to determine the cellular source of regenerated CMs [5, 43–50]. Cardiac stem cells, such as c-kit-positive CPCs, islet 1-positive CPCs, stem cell antigen-1-positive CPCs, and cardiosphere-derived cells, have attracted considerable attention as cellular sources of regenerated CMs in the 2000s [44–47]; further, clinical trials using cardiac stem cells in patients with LV dysfunction have been conducted (Table 1). In the prospective, randomized CArdiosphere-Derived aUtologous stem CElls to reverse ventricUlar dySfunction (CADUCEUS) trial, an intracoronary infusion of cardiosphere-derived cells 1.5–3 months after MI reduced cardiac scar size; however, it did not improve LV systolic function after 1 year [51, 52]. In the Stem Cell Infusion in Patients with Ischemic cardiOmyopathy (SCIPIO) trial, post-MI patients with LV dysfunction who underwent coronary artery bypass grafting (CABG) were assigned to receive treatment with an intracoronary infusion of autologous c-kit-positive CPCs 4 ± 1 months after CABG. An intracoronary infusion of c-kit-positive CPCs effectively improved the LV systolic function and reduced the infarct size in these patients [53]. However, it is unclear whether c-kit-positive CPCs efficiently transdifferentiated into functional CMs [44, 54, 55]. To examine this possibility, Molkentin et al. performed lineage-tracing analysis after the labeling of c-kit-expressing cells in adult mice and demonstrated that the number of c-kit-positive cells that transdifferentiated into new CMs was low (<0.03%) even after cardiac injury, indicating that c-kit-positive CPCs are not a major source of newly generated CMs [56].

**Table 1:** Clinical trials using cardiac stem cells

Trial			CADUCEUS	SCIPIO
Inclusion criteria	Patient characteristics		Previous MI	Previous MI and CABG
	EF (%)		25–45	≤40
No. of patients	Total		25	23
	Cell therapy group		17	16
	Control group		8	7
Cell therapy	Type of cardiac stem cells		Cardiosphere-derived cells	c-kit-positive CPCs
	Dose of injected cells		12.5–25 million	0.5–1 million
	Delivery method		Intracoronary infusion	Intracoronary infusion
	Timing of delivery		1.5–3 months after MI	4 ± 1 months after CABG
Outcomes	EF (%); baseline/follow-up	Cell therapy	42.4/48.2 (1 year)	30.3/38.5 (4 months)
		Control	42.5/48.1 (1 year)	30.1/30.2 (4 months)
	Scar size (%LV or g); baseline/follow-up	Cell therapy	23.8/12.9 (1 year) (%LV)	32.6/22.8 (1 year) (g)
		Control	22.4/20.3 (1 year) (%LV)	N/A
References			[51, 52]	[53]

*CABG* coronary artery bypass grafting;, *CPCs* cardiac progenitor cells, *EF* ejection fraction, *LV* left ventricular, *MI* myocardial infarction, *N/A* not available

Recent genetic fate-mapping experiments revealed that the regeneration of CMs occurs by the division of pre-existing CMs during normal aging at a low rate and that this process is enhanced in response to cardiac injury [5]. Therefore, it is accepted that new CMs are primarily derived from the division of pre-existing CMs. However, it remains unclear what prevents cell division in adult mammalian CMs whose endogenous regenerative capacity is insufficient to restore cardiac function after substantial damage. Therefore, growth factors, transcription factors, microRNAs, and small molecules that stimulate CM replication have been actively studied (Table 2) [37, 57–67]. Furthermore, the roles of the physiological and pathological environments of the heart in the regulation of cardiac regeneration have been studied with great detail (Fig. 1).

**Table 2:** Growth factors, transcription factors, microRNAs, and small molecules that stimulate CM replication

		Models/treatments		Species	Age or weight/cells	Follow-up	Markers of proliferation	Functional improvements comments	References
Factors	Neuregulin 1	In vitro		Rat	ARCM		BrdU, Aurora B		[57]
		In vivo	IP injection	Mouse	12 weeks	9 days	BrdU, pH3, Aurora B		
		MI	IP injection after MI	Mouse	8 weeks	14 weeks	BrdU, pH3, Aurora B	EF, scar size	
	Periostin	In vitro		Rat	ARCM		BrdU, Aurora B		[58]
		In vivo	Injection into the myocardium	Rat	300 g	7 days	BrdU, Aurora B		
		MI	Gelfoam patches to epicardial after MI	Rat	300 g	12 weeks	BrdU, Aurora B (1 and 12 weeks)	EF, FS, scar size	
	Oncostatin M	In vitro		Rat	NRCM		EdU		[59]
		MI	IP injection after MI	Mouse	12 weeks	21 days		survival, EF	
	Salvador (Salv)	In vivo	*NKX 2.5*-*Cre*; *Salv* ^*flox*/*flox*^ (*Salv CKO*)	Mouse	E12.5, P2		pH3 (E12.5)	Thickened ventricular walls, enlarged ventricular chambers	[60]
	Yes-associated protein (Yap)	In vivo	α*MHC*-*YapS112A*	Mouse	P28	21 days	pH3, Aurora B (7 days)	FS, scar size (21 days)	[61]
	Yes-associated protein (Yap)	In vivo	α*MHC*-*rtTA*; *Yap*, *induction by doxycycline*	Mouse	8 weeks	5 weeks	EdU, pH3	EF, scar size	[62]
		In vivo	AAV9-cTnT-hYap injection into the myocardium	Mouse	10–12 weeks	23 weeks	EdU (5 days)	FS (4 weeks), survival (23 weeks)	
miRNAs	miR-15 family	In vivo	anti-miR-15/16 s.c. injection	Mouse	P2	10 days	pH3		[63]
	miR-15 family	I/R	anti-miR-15 s.c. injection before I/R	Mouse	P21	21 days	pH3 (7 days)	FS (21 days)	[37]
	miR-590, miR-199a	In vitro	Transfection	Rat/Mouse	ARCM, NRCM, NMCM		Ki67, EdU, pH3, Aurora B		[64]
		In vivo	hsa-miR-590-3p or hsa-miR-199a-3p injection into the myocrdium	Rat	P0	4 days	EdU		
		In vivo	AAV9-miR-590 or AAV9-miR-199a IP injection	Mouse	P0	12 days	pH3		
		MI	AAV9-miR-590 or AAV9-miR-199a IP injection after MI	Mouse	8–12 weeks	60 days	EdU	EF, FS, scar size	
	miR-222	In vitro	Transfection	Rat	NRCM		Ki67, EdU		[65]
		I/R	α*MHC*-*tTA*; *mi*R-222 induction by doxycycline deprivation before I/R	Mouse	10–12 weeks	6 weeks	EdU. pH3	FS, scar size	
	miR-17-92 family	In vitro	Transfection	Rat	NRCM		EdU, Aurora B		[66]
		In vivo	*NKX 2.5*-*Cre*; *miR*-*17*–*92*	Mouse	E16.5, P4		pH3		
		In vivo	α*MHC*-*Cre*; *miR*-*17*–*92*	Mouse	P15		EdU, pH3, Aurora B		
		MI	αMHC-MerCreMer; miR-17–92 induction by tamoxifen after MI	Mouse	2 months	4 months	EdU	FS, scar size	
Small molecule	BIO	In vitro		Rat	NRCM		BrdU, pH3		[67]

*AAV* adeno-associated virus, *ARCM* adult rat cardiomyocytes, *BrdU*, 5-bromo-2′-deoxyuridine, *EdU* 5-ethynyl-2′-deoxyuridine, *EF* ejection fraction, *FS* fractional shortening, *IP* intraperitoneal, *I/R* Ischemia/reperfusion, *MI* myocardial infarction, *NMCM* neonatal mouse cardiomyocytes, *NRCM* neonatal rat cardiomyocytes, *pH3* Phospho-Histone H3; *s.c.* subcutaneous

### Impact of reactive oxygen species on CM regeneration

Recently, considerable attention has been given to the impact of reactive oxygen species (ROS) on cardiovascular diseases. Cardiac injury has been shown to increase the amount of ROS in the heart, which induces CM cell cycle arrest via the activation of responses to DNA damage (Fig. 2) [68, 69]. The inhibition of ROS by pretreatment with N-acetyl-L-cysteine has been shown to promote CM regeneration after ischemia–reperfusion injury even in 21-day-old mice [69]. In addition, the presence of oxygen in the environment has been reported to influence the production or scavenging of ROS and regeneration of CMs. Hyperoxic (100% oxygen) and hypoxic (15% oxygen) environments have been found to diminish and enhance CM proliferation, respectively, in neonatal mice with adequate CM regenerative capacities (Fig. 2) [69]. Furthermore, in adult mice, gradual exposure to severe hypoxia after MI, in which inspired oxygen is gradually decreased by 1% beginning 1 week after MI for 2 weeks, and then maintained at 7% for another 2 weeks, has been found to induce CM regeneration and coronary angiogenesis, resulting in improvements in the LV systolic function [70]. To evaluate the proliferation of hypoxic CMs in the adult heart, hypoxic CMs in α*MHC*-*creERT2*-*ODD*; *R26R*/*tdTomato* mice were genetically labeled at 2 months of age and fate mapped for 1 month under normal conditions; the results of this study demonstrated that labeled hypoxic CMs have a higher proliferative capacity than unlabeled CMs and can be a source of newly generated CMs [71].

### Role of macrophages in cardiac regeneration

One-day-old mice can completely regenerate their hearts after MI injury. However, 14-day-old mice do not retain sufficient capacity for cardiac regeneration and cause fibrosis in response to cardiac injury. Clodronate liposome-mediated depletion of monocytes/macrophages in 1-day-old mice after MI reduces the angiogenic response, blocks the cardiac regenerative capacity, and induces cardiac fibrosis and dysfunction [72]. To identify the role of cardiac monocytes/macrophages in cardiac regeneration, immunophenotyping and gene expression profiling of cardiac monocytes/macrophages from 1-day-old and 14-day-old mice were isolated and compared after MI [72]. Regenerative macrophages from 1-day-old mice displayed both M1- and M2-associated gene transcription patterns and expressed more chemokines, proangiogenic factors, and oxidative stress responders, which may facilitate the formation of new myocardium than macrophages from 14-day-old mice.

**Fig. 3:**
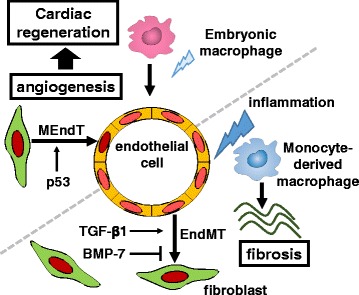
Angiogenic and fibrogenic responses during cardiac tissue injury and repair. Both MEndT and EndMT actively contribute to cardiac angiogenesis and fibrosis after cardiac injury. Embryonic macrophages can promote angiogenesis and subsequent cardiac regeneration in neonatal mice after cardiac injury, but infiltrate macrophages during adult cardiac injury do not

Embryonic-derived resident cardiac macrophages (MHC-II^low^CCR2^−^) and two types of resident cardiac macrophages (MHC-II^low^CCR2^−^ and MHC-II^high^CCR2^−^) are the major populations of monocytes/macrophages in neonatal and adult mouse hearts, respectively; monocytes (MHC-II^low^CCR2^+^) and monocyte-derived macrophages (MHC-II^high^CCR2^+^) are not abundant in either neonatal or adult hearts under normal physiological conditions [73]. To elucidate essential cardiac monocyte/macrophage subsets involved in cardiac regeneration, Lavine et al. used a diphtheria toxin receptor-mediated CM ablation mouse model [73], in which cardiac injury was induced without concomitant systemic inflammation. In response to diphtheria toxin receptor-mediated cardiac injury, the neonatal heart selectively expanded the population of embryonic-derived resident cardiac macrophages and cardiac dysfunction recovered to baseline. In contrast, in adult mice, the heart recruits CCR2^+^ pro-inflammatory monocytes and monocyte-derived macrophages and loses CCR2^−^ resident cardiac macrophages after cardiac injury; cardiac function recovery was not observed. However, the administration of selective CCR2 inhibitors in adult mice after cardiac injury inhibited CCR2^+^ monocyte recruitment to the heart and preserved CCR2^−^ resident cardiac macrophages, resulting in reduced inflammation and enhanced angiogenesis. Collectively, embryonic-derived resident cardiac macrophages are key mediators of angiogenesis, leading to cardiac regeneration in response to cardiac injury (Fig. 3).

### Interactions between endothelial cells and fibroblasts

EndMT is a fundamental cellular mechanism that regulates embryonic development and fibrotic diseases. During the embryonic development of the heart, the endocardium undergoes EndMT and forms an atrioventricular cushion: the primordial valves and septa of the adult heart [74]. Zeisberg et al. demonstrated that Tie1-expressing endothelial cells in the adult heart underwent EndMT and differentiated into fibroblasts during cardiac fibrosis in response to pressure overload [15]. Endothelial cells undergoing EndMT lost tight junctions that hold neighboring cells, gained the ability to move, and contributed to the total pool of cardiac fibroblasts. Although endothelial cells are not major origins of cardiac fibroblasts under normal conditions, inflammation induces EndMT of endothelial cells. As a result, approximately one-third of all cardiac fibroblasts originated from endothelial cells in the fibrotic heart in response to pressure overload. During this process, TGF-β1 induces EndMT, whereas bone morphogenic protein 7 (BMP-7) prevents EndMT and preserves the endothelial phenotype [15]. Therefore, the anti-fibrotic effects of recombinant human BMP-7 have been investigated. An intraperitoneal injection of recombinant human BMP-7 inhibited EndMT and the progression of cardiac fibrosis and improved diastolic cardiac function in a moderate-intensity pressure overload model. Furthermore, the inhibition of EndMT and cardiac fibrosis by recombinant human BMP-7 has been observed in a mouse model of chronic heart rejection caused by heterotopic heart transplantation with a class II major histocompatibility mismatch between donor and recipient [15].

Cardiac fibroblasts can undergo mesenchymal-to-endothelial transition (MEndT) immediately after ischemic cardiac injury [75]. Approximately 30% of fibroblasts in the injury zone undergo MEndT, and fibroblast-derived endothelial cells exhibit anatomical and functional characteristics of native endothelial cells and contribute to angiogenesis of the injured heart. p53, a transcription factor, regulates MEndT in cardiac fibroblasts [75]. The loss of p53 in Col1a2-expressing fibroblasts severely decreases the formation of fibroblast-derived endothelial cells, reduces the post-MI vascular area, and worsens cardiac function. Conversely, the stimulation of the p53 pathway after ischemic cardiac injury by an intraperitoneal injection of the small molecule: reactivation of p53 and induction of tumor cell apoptosis (RITA), which inhibits ubiquitin-mediated p53 degradation, augments MEndT, enhances angiogenesis, and improves cardiac function. However, although cardiac fibroblasts cultured in vitro under serum-free conditions have been found to form tubular structures resembling the endothelial cell architecture and express endothelial markers, cardiac fibroblasts cultured under serum-fed conditions fail to generate tubular structures, even when p53 is artificially overexpressed. This result suggests that p53 expression alone is insufficient to induce MEndT and that the microenvironment, growth factors, and other signals are involved in this process. Collectively, these close interactions between endothelial cells and fibroblasts regulate cardiac fibrosis and angiogenesis (Fig. 3), and the regulation of both EndMT and MEndT is a potential therapeutic target for enhancing cardiac repair.

### Direct reprogramming of cardiac fibroblasts into CMs

In 2006, Takahashi and Yamanaka generated induced pluripotent stem (iPS) cells from mouse fibroblasts by introducing four factors: Oct3/4, Sox2, c-Myc, and Klf4 [76]. Subsequently, the direct reprogramming of fibroblasts by lineage-specific transcription factors into the primary functional cells of each organ, such as neurons, hepatocytes, and renal tubular epithelial cells, was accomplished [77–80]. Further, the direct reprogramming of mouse cardiac fibroblasts into CMs is induced by a combination of cardiac-specific transcription factors (Gata4, Mef2c, and Tbx5) in vitro [81]. Furthermore, endogenous cardiac fibroblasts were directly reprogrammed into CMs by the retrovirus-mediated delivery of cardiac-specific transcription factors in vivo, with such newly generated CMs reducing scar formation and cardiac dysfunction after MI [49, 50]. Several laboratories demonstrated that in vivo reprogramming yields higher quality of CMs than in vitro reprogramming. These results suggest that factors within the native microenvironment, such as the ECM, growth factors, local signals, and mechanical forces, enhance the maturity of CMs in the heart.

Although the direct reprogramming of cardiac fibroblasts into CMs in vivo can be a new cardiac regenerative therapy (Fig. 2), the efficiency of reprogramming is currently low to adequately improve cardiac function, and the mechanisms of reprogramming and properties of newly generated CMs have not yet been fully defined [82]. Therefore, the modification of transcription factors and induction of microRNAs have been studied, with the goal of improving the quality of cardiac reprogramming [50, 83]; the addition of factors that regulate the native microenvironment may enhance the efficacy of cardiac direct reprogramming.

## Conclusions

Most types of cardiac cells, including cardiac fibroblasts, CMs, macrophages, and endothelial cells, regulate cardiac fibrosis in a coordinated manner; therefore, various elements and signals could be therapeutic targets for cardiac protection and the prevention of cardiac fibrosis. We commonly use ACE inhibitors or ARBs to block RAS signaling and inhibit cardiac fibrosis in patients with hypertension and cardiac diseases; however, there are few effective therapies that target other pathways involved in the prevention of cardiac fibrosis. Although targeting TGF-β signaling is a promising strategy, optimizing the appropriate timing and duration of treatment remains a challenging task.

Recently, it has been revealed that inflammatory and fibrotic responses to myocardial damage are essential for cardiac repair as well as cardiac regeneration; paracrine factors, the microenvironment, and small molecules that regulate these processes are all currently under investigation. Non-CMs, including macrophages, fibroblasts, and endothelial cells, cooperate with CMs to promote cardiac repair and regeneration. The regulation of the hypoxic environment and macrophage polarization may enhance crucial angiogenic responses involved in these processes. Further, the direct reprogramming of cardiac fibroblasts into functional CMs is an attractive strategy, and currently, investigations to improve the efficiency and maturity of generated CMs are in progress. Further research to unravel the regulatory mechanisms underlying cardiac fibrosis and regeneration will aid the development of therapeutic strategies to treat and prevent advanced heart failure.

## Abbreviations

AAV: Adeno-associated virus
ACE: Angiotensin-converting enzyme
Ang II: Angiotensin II
ARB: Angiotensin receptor blocker
ARCM: Adult rat cardiomyocytes
AT1: Ang II type 1
BMP-7: Bone morphogenic protein 7
BrdU: 5-bromo-2′-deoxyuridine
CABG: Coronary artery bypass grafting
CM: Cardiomyocyte
CPCs: Cardiac progenitor cells
ECM: Extracellular matrix
EdU: 5-ethynyl-2′- deoxyuridine
EF: Ejection fraction
EndMT: Endothelial-to-mesenchymal transition
FS: Fractional shortening
I/R: Ischemia/reperfusion
IGF-1: Insulin-like growth factor-1
IP: Intraperitoneal
KLF5: Krüppel-like factor 5
LV: Left ventricular
MEndT: Mesenchymal-to-endothelial transition
MI: Myocardial infarction
N/A: Not available
NMCM: Neonatal mouse cardiomyocytes
NRCM: Neonatal rat cardiomyocytes
PDGF-A: Platelet-derived growth factor-A
PEO: Proepicardial organ
pH3: Phospho-Histone H3
RAS: Renin–angiotensin system
RITA: Reactivation of p53 and induction of tumor cell apoptosis
ROS: Reactive oxygen species
s.c.: Subcutaneous
TGFBR1: TGF-β type I receptor
TGF-β: Transforming growth factor-beta
